# Prefrontal cortex neural activity predicts reduction of non-suicidal self-injury in adolescents with major depressive disorder: An event related potential study

**DOI:** 10.3389/fnins.2022.972870

**Published:** 2022-11-03

**Authors:** Huishan Liu, Yujiao Wen, Xiumei Liang, Yifan Xu, Dan Qiao, Chunxia Yang, Min Han, Hong Li, Tian Ren, Xuemin Zhang, Gaizhi Li, Zhifen Liu

**Affiliations:** Department of Psychiatry, The First Hospital of Shanxi Medical University, Taiyuan, China

**Keywords:** non-suicidal self-injury behavior, adolescent, major depressive disorder, ERP, P300

## Abstract

**Background:**

Non-suicidal self-injury (NSSI) is common in adolescent MDD, which is also a risk factor for suicide. However, there is few research on biomarkers and predictors about treatment response of NSSI. The purpose of this study was to find the difference of P300 between adolescent MDD with NSSI and healthy controls, and to explore whether the baseline electrophysiological level can predict the change of NSSI after treatment.

**Methods:**

We collected 62 first-episode drug-naïve MDD adolescents with NSSI (MDD with NSSI group) and 44 healthy controls (HC group). The demographic data, HAMD score, self-injury frequency and electrophysiological level of NSSI group and HC group were collected. The HAMD score, frequency of NSSI in was also collected after 8 weeks of antidepressant treatment.

**Results:**

Compared to HC, the latency of the N2, P3a, and P3b components were significantly prolonged, whereas the amplitude of P3a and P3b were decreased in the MDD with NSSI group (*P* < 0.001). The frequency of self-injury decreased significantly after treatment (*P* < 0.001). Regression analysis showed that the amplitudes of P3b had a significant positive predictive effect on the rate of change of NSSI frequency after 8 weeks.

**Conclusion:**

P3b at baseline can be used as potential predictor for the reduction of NSSI in adolescent MDD.

## Introduction

Non-suicidal self-injury (NSSI) behavior refers to those behaviors that directly and intentionally damage one’s body without the purpose of suicide, and is socially and culturally unacceptable (Ross and Heath, 2002). Common forms of NSSI include pulling hair, scalding, cutting skin, scratching, hitting oneself, preventing wounds from healing, biting, pricking needles, and swallowing dangerous substances, et al. (Saraff and Pepper, 2014). NSSI behavior is listed as an independent clinical disorder in the Diagnostic and Statistical Manual of Mental Disorders, 5th Edition (DSM-5) (Andover, 2014; Zetterqvist, 2015).

Adolescent with NSSI behavior is common in the world, and its incidence is increasing year by year. About 14–15% of global adolescents have experienced NSSI behavior at least once (Liu et al., 2018). According to the results of a survey, 13.5% of girls and 4.3% of boys aged 14–17 said they had experienced NSSI at least once in their lifetime. The incidence of NSSI behaviors varies in different countries. The incidence of NSSI behaviors is 13.8% in Scotland, 15.3% in the United States and 24% in New Zealand, and only 3.1% in Germany. Adolescent in different regions of China have different degrees of NSSI behaviors, and the incidence is gradually increasing (Whitlock et al., 2011; Fleming et al., 2014; Rasmussen and Hawton, 2014; Cimen et al., 2017; Zhang et al., 2018).

Investigations have found that adolescent with major depressive disorder (MDD) are prone to risk behaviors such as self-injury and suicide (Jacobson and Gould, 2007), NSSI may be a unique and important risk factor for suicide (Klonsky et al., 2013). Suicide is the third major cause of death among adolescents (Centers for Disease Control and Prevention [CDC], 2009). The detection rate of suicidal ideation among adolescents is 10.72–12.1%, and that there are suicide attempts and plans for adolescents is 8.1% (Laye-Gindhu and Schonert-Reichl, 2005). In addition, NSSI is also common among adolescents with MDD, but little attention had been paid to it in clinical studies. NSSI will have a great impact on adolescents and seriously harm their physical and mental health.

Treatment for adolescents MDD with NSSI behavior include drug therapy, psychotherapy, physical therapy, combined therapy, etc. A non-RCT study found that ziprasidone was effective in reducing the incidence of NSSI behavior in adolescents compared with risperidone, olanzapine, and promethazine (Libal et al., 2005). A systematic review indicated the effectiveness of dialectical behavioral therapy, cognitive behavioral therapy, and psychosocial basic therapy in the treatment of adolescent NSSI (Ougrin et al., 2014). Sertraline is one of the first selective serotonin reuptake inhibitors (SSRIs) approved for the treatment of childhood and adolescent depression, and it is also the most widely used drug (Gómez-Lumbreras et al., 2021). It can effectively relieve the depressive symptoms in a short time, improve the cognitive function of the patients, and improve the quality of life of the patients (Kaštelan et al., 2019). Currently, there are a variety of treatment methods for adolescent with MDD with NSSI behavior, however, the treatment response varies, so it is critical to find an effective predictor of treatment response.

Previous studies on NSSI behavior mostly focused on emotion regulation, ignoring the role of cognition. On the basis of summarizing four emotion regulation models, Hasking et al. (2017) combined cognitive model and emotion model to construct a new cognitive emotion model related to NSSI, which reflects the important role of cognition. At present, the research on cognitive factors of self-injury behavior mostly adopts neuropsychological test or scale, and research on objective predictors are still lacking. Event related potential (ERP) is a suitable choice due to its high time resolution, simplicity, convenience and cheapness. One component related to cognitive function is P300, which is considered to reflect cognitive processes, including attention distribution, executive function and memory (Polich, 2012). The cerebral cortex of suicidal depressed patients showed a decrease in serotonin-activated neurological function and a significant increase in the amplitude of prefrontal P300, so P300 can be regarded as a reference index to predict the risk of suicide in suicidal depressed patients (Chen et al., 2005). The previous research group found that, compared with the HC group, the adolescent with MDD with NSSI behavior significantly prolonged the incubation period on P300, significantly reduced the amplitude, and had significant cognitive dysfunction, such as executive dysfunction and memory impairment (Wen et al., 2021). Based on this, sertraline was selected as the treatment drug in this study. The incubation period and amplitude of P300 at baseline were used as predictors, and the scores of Hamilton Depression Scale (HAMD) and NSSI Diary Card at baseline and 8 weeks were used as indicators. To observe whether the changes of ERP can predict the clinical efficacy of sertraline in the treatment of adolescent with MDD with NSSI behavior.

The purposes of this study are: firstly, to explore the difference between NSSI group and HC group by the related indicators of ERP, HAMD scores and the NSSI frequency; secondly to examine whether the changes of ERP can predict the clinical efficacy of sertraline in the treatment of adolescent MDD with NSSI.

## Materials and methods

### Participants

The study included 106 subjects aged 10–23 years: 62 unmedicated patients with first-episode adolescent MDD with NSSI and 44 healthy control (HC) subjects. All NSSI group patients were from the Department of psychiatry and mental health, the First Hospital of Shanxi Medical University. All HC group subjects were recruited from Taiyuan City, Shanxi Province, China, using community advertisements. All subjects were independently evaluated by two trained psychiatrists using structured clinical interviews for DSM-5, Research Version (SCID-5-RV). The Research Ethics Committee of the First Hospital of Shanxi Medical University approved this study.

#### Inclusion and exclusion criteria for patients with MDD

The inclusion criteria for MDD patients were as follows: (1) age between 10 and 23 years with no restrictions on gender; (2) DSM-5 diagnostic criteria for MDD; (3) right handed; (4) first-episode MDD with no previous use of antidepressant or other psychotropic medications; and (5) volunteered to participate in the study and signed the informed consent form. The exclusion criteria were as follows: (1) patients with severe or unstable heart, liver, kidney, endocrine, blood and other internal diseases and nervous system diseases; (2) any cooccurring mental disorder; (3) alcohol dependence or abuse; (4) previous history of nervous system disease or brain injury; (5) personal or family history of epileptic seizures; (6) other situations that are not suitable to participate in this study.

#### Inclusion and exclusion criteria for HCs

Inclusion criteria for HCs were as follows: (1) age 10–23 years; (2) no mental disorder found in the initial screening; (3) matched to the MDD patients in terms of sex and education level; and (4) participated voluntarily and signed the informed consent form. The exclusion criteria were as follows: (1) organic disease; (2) alcohol abuse within 30 days or alcohol or drug dependence within 6 months prior to the screening; (3) participation in other clinical trials in the previous 3 months; and (4) other conditions that disqualified the subject from the study, as determined by the investigators.

### Measures

Eligible participants were asked to provide sociodemographic information including name, gender, age. For clinically related variables measures, we used the Hamilton depression scale-24 (HAMD-24) to assess the severity of depressive symptom. NSSI Diary Card was used to record the frequency of NSSI. Most items in HAMD-24 adopt a 5-level scoring method of 0–4 points. The criteria at all levels are: 0-none, 1-mild, 2-moderate, 3-severe, 4-extremely severe. A few items adopt a three-level scoring method of 0–2 points, and the grading standards are: 0-none, 1-mild-moderate, and 2-severe. If the total score exceeds 35, it may be severe depression; More than 20 points may be mild or moderate depression; If less than 8 points, there is no depressive disorder. NSSI diary card was used to record the number of self-injuries in the past month and 1 week.

For eligible adolescents with MDD, sertraline was used for treatment, with a daily dose of 50–200 mg and an initial dose of 25–50 mg.

### Event related potential parameters

Event related potential data were collected using the 128-electrode NEMUS 2 system (Brain products GmbH, Germany). Recording electrodes were placed at the Fz, Cz, and Pz positions; the electrode at the Cz position was the standard and those at the Fz and Pz positions were references for waveform identification. Reference electrodes are TP9 and TP10, and the ground electrode (GND) are placed in the middle of the parietal lobe.

#### P300 detection

The task employed the classic Oddball experimental paradigm. The stimulus sequence was composed of a target stimulus (T) and non-target stimulus (NT) at a probability ratio of 0.2/0.8; T was randomly interspersed among NT, and the task consisted of 60 T and 240 NT. Subjects were required to press a key as soon as T appeared. The stimulus frequency was 0.5–1 time/s; stimulus interval was 1–3 s; and total task duration was 14 min. Electrode resistance was <5 kΩ; the time window for data segmentation was -200 to 1500 ms.

### Statistical analysis

Data were analyzed using SPSS 22.0 (SPSS Inc., Chicago, IL, USA). The threshold of statistical significance was set as α = 0.05 for all the analyses. For the demographic data, categorical variables were compared with the χ*^2^* test and continuous variables were compared using the two independent sample *t* test, which was used for HAMD-24, NSSI diary card scores. Mann-Whitney *U* test was also used to analyze ERP indicators, the major components of ERPs were identified and their index values determined according to the internationally recognized maximum waveforms of the time analysis window. Linear regression analysis was used to predict the improvement of depression and NSSI frequency in the NSSI group. The results were considered significant if *P* < 0.05, corrected by false discovery rate (FDR).

## Results

### Demographics and clinical characteristics of all participants

There were no significant differences between the two groups in terms of age, gender, and education years (*P* > 0.05). The NSSI and HC groups showed significant differences in HAMD-24 and NSSI frequency (both 1 mouth and 1 week) (*P* < 0.001) (Table 1).

**TABLE 1:** Demographic and clinical of all participants.

Variable	MDD with NSSI (*n* = 62)	HC (*n* = 44)	χ*^2^/F/t*	*P*
Gender				
Male	16	11	0.009	0.925
Female	46	33		
Age, years	16.74 ± 2.72	17.34 ± 2.85	–1.093	0.277
Education, years	9.66 ± 2.61	10.34 ± 2.92	–1.254	0.213
HAMD-24	25.74 ± 6.35	2.00 ± 2.83	23.18	<0.001***
NSSI diary card (1 mouth)	2.44 ± 1.78	0.00 ± 0.000	9.96	<0.001***
NSSI diary card (1 week)	1.52 ± 1.91	0.00 ± 0.000	5.803	<0.001***

***Indicates *p* < 0.001.

All subjects were students of Han ethnicity, not married, with no religious affiliation.

Data represent number, mean ± standard deviation.

HAMD, Hamilton Depression Scale; HC, healthy control; NSSI, non-suicidal self-injury.

The frequency of NSSI in the NSSI group in the past 1 month was assessed at baseline (0 week) using NSSI diary card.

The frequency of NSSI in the NSSI group in the past 1 week was assessed at baseline (0 week) using NSSI diary card.

### Event related potential results analysis between the two groups

**FIGURE 1:**
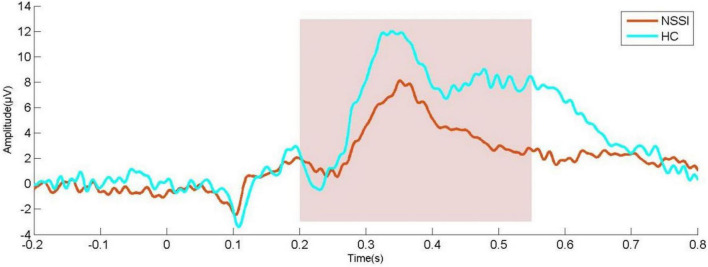
Waveform diagram of P300 in CZ channel; NSSI, non-suicidal self-injury (*n* = 62); HC, healthy control (*n* = 44).

**FIGURE 2:**
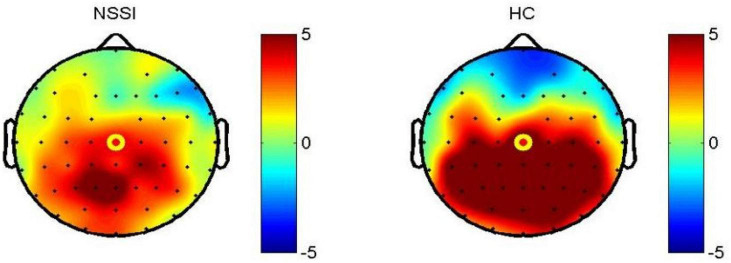
Topographic map of P300 in CZ channel 200–500 ms; NSSI, non-suicidal self-injury (*n* = 62); HC, healthy control (*n* = 44).

Compared with HC subjects, the latency of N2, P3a, and P3b in NSSI group were significantly prolonged; the amplitude of P3a and P3b decreased (*P* < 0.001). In other ERP components, there was no significant difference between the two groups (Table 2 and Figures 1, 2).

**TABLE 2:** P300 value between the NSSI and HC groups.

P300		NSSI	HC	*Z*	*P*
			
		Md (P25, P75)	Md (P25, P75)		
Latency, ms	N1	105.5 (99.25, 119)	109 (102.75, 118)	–0.712	0.476
	P2	206.5 (200, 220.75)	202 (195.75, 208.75)	–1.745	0.135
	N2	243.5 (231.25, 258.75)	232 (218, 252.25)	–2.318	0.04*
	P3a	343 (332, 356)	316 (306.75, 332.25)	–5.668	<0.001***
	P3b	370.5 (363.25, 386)	331 (323, 345)	–7.638	<0.001***
Amplitude, μV	N1	−2.81 (−6.12, 0.52)	−3.425 (−5.795, −1.44)	–0.933	0.39
	P2	2.195 (0.15, 4.42)	3.18 (0.325, 5.86)	–1.064	0.36
	N2	−0.3 (−3.385, 3.38)	−0.91 (−4.22, 0.81)	–1.363	0.25
	P3a	7.47 (3.725, 11.58)	11.76 (9.58, 14.25)	–4.29	<0.001***
	P3b	8.315 (3.45, 11.26)	12.76 (9.36, 14.73)	–4.796	<0.001***

*Indicates *p* < 0.05; ***indicates *p* < 0.001.

Data represent Median (quartile, third quartile).

ERP, event-related potential; HC, healthy control; NSSI, non-suicidal self-injury.

### Changes of clinical symptoms after treatment

The results of paired sample *t*-test showed that HAMD-24 total score and the frequency of NSSI decreased significantly after 8 weeks of treatment (Table 3).

**TABLE 3:** NSSI frequency and HAMD-24 score before and after treatment.

	Baseline	8 week	*t*	*P*
HAMD-24	25.74 ± 6.35	12.11 ± 6.75	12.012	<0.001***
NSSI diary card (1 mouth)	2.44 ± 1.78	0.08 ± 0.33	9.962	<0.001***
NSSI diary card (1 week)	1.52 ± 1.91	0.08 ± 0.33	5.619	<0.001***

***Indicates *p* < 0.001.

### Regression analysis of event related potential index and clinical symptom improvement

Taking the latency and amplitude of N1, P2, N2, P3a, and P3b and total score of HAMD-24 after 8 weeks as independent variables and the rate of change of NSSI frequency between 8 weeks and baseline as dependent variables, the linear regression results show that:

In the assessment of NSSI frequency, the amplitude of P3b (*t* = 5.242, *P* < 0.001) has significant positive prediction effect on the change of NSSI frequency. Other indexes in the independent variable have no significant difference in the prediction effect on the dependent variable (Table 4).

**TABLE 4:** Regression analysis of the reduction rate of NSSI after 8 weeks of treatment.

Variable		B	B 95% CI		Beta	*t*	*P*
Latency, ms	N1	–0.001	–0.014	0.012	–0.025	–0.191	0.849
	P2	0.001	–0.01	0.019	0.230	1.917	0.063
	N2	0.000	–0.08	0.007	–0.006	–0.061	0.952
	P3a	0.002	–0.014	0.018	0.045	0.275	0.785
	P3b	0.002	–0.012	0.017	0.055	0.328	0.745
Amplitude, μV	N1	–0.029	0.073	0.014	–0.164	–1.372	0.178
	P2	–0.043	–0.09	0.04	–0.195	–1.852	0.072
	N2	0.02	–0.014	0.18	0.109	0.957	0.785
	P3a	–0.011	–0.046	0.024	–0.077	–0.62	0.539
	P3b	0.112	0.069	0.155	0.672	5.242	<0.001***
HAMD-24 (8 week)		–0.015	–0.039	0.010	–0.126	–1.234	0.225

R^2^ = 0.666, *F* = 6.693, *df* = 11, *P* < 0.001.

***Indicates *p* < 0.001.

*R*^2^, coefficient of determination; F, statistics in F, Fisher–Snedecor test; df, degrees of freedom; P, probability in the test; B, unstandardized parameter; CI, confidence interval; Beta standardized parameter (size of effect); t, statistics in *t* test.

NSSI, non-suicidal self-injury.

## Discussion

In this study, 62 first-episode adolescent MDD with NSSI behavior and 44 healthy controls were included. The oddball task was used to observe the difference of electrophysiological level between the two groups. Regression analysis was used to explore whether baseline P300 can be used as a neural marker to predict the clinical efficacy of sertraline tablets in the treatment of adolescent MDD with NSSI behavior.

In this study, the MDD adolescents with self-injury behavior is mainly in the middle school stage, which is basically consistent with previous research results (Swannell et al., 2014; Gao et al., 2021; Jiang et al., 2021).

P300 incubation period is an electrophysiological index reflecting the speed of mental activities, and its amplitude can reflect the utilization of effective resources by the brain in information processing, which mainly depends on the sensitivity of the patient to stimulation (Duncan et al., 2005). This study found that compared with HC group, NSSI group had significantly longer N2, P3a, and P3b latency and significantly lower P3a and P3b amplitude, indicating that patients with depression had lower brain nerve excitability and cognitive speed, suggesting that NSSI group may have cognitive impairment. This is consistent with previous study, as Zhou et al. (2022) used oddball paradigm to compare the differences of P3b components among NSSI + MDD, MDD and HC groups. Leone et al. (2021) used laser evoked potential as an index to study the suicide risk of NSSI adolescents suggest that the amplitude of N2 component in NSSI patients is reduced, which is different from our study, may be due to different experimental paradigms. In this study, no significant difference was found in the latency of N1 between the two groups, which was different from Wen’s study (Wen et al., 2021). Which may be related to the age of healthy subjects. Some studies suggest that N2 represents reaction inhibition and conflict monitoring, P3a is related to automatic attention capture, and P3b is related to stimulus classification and processing, working memory, reaction inhibition and executive function (Bareš et al., 2007; Albert et al., 2010; Sanger and Dorjee, 2015; Deiber et al., 2021; Penengo et al., 2022), which seems to explain the results of this study. One study found that the main effect of N2 component was significant under whether self-injury cues were present, and N2 represented conflict detection and monitoring, which may indicate that greater conflicts were generally detected during exposure to self-injury cues (Zhou et al., 2022). Under the self-injury cue, the P3 amplitude of NSSI group was larger than that of HC group, and the P3 amplitude with the self-injury cue was significantly larger than that with the neutral cue, indicating that the neural response of NSSI adolescents changed during exposure to the self-injury cue (Zhou et al., 2022). Allen found difficulty in response inhibition in the group of eating disorders with NSSI (Allen et al., 2020), Nilsson also found that compared with healthy people (Nilsson et al., 2021), patients with intentional self-mutilation had defects in cognitive flexibility and response inhibition, and Zhang also found executive dysfunction in the group of MDD adolescents with NSSI (Zhang et al., 2022). Our study find neuroelectrophysiological evidence in the adolescent MDD with NSSI compared with HC.

At present, there is no effective drug treatment option for adolescent NSSI. According to the treatment guidelines for adolescent MDD (Cheung et al., 2018; American Psychological Association [APA], 2019) and the treatment guidelines for adolescent NSSI (National Institute for Health and Care Excellence [NICE], 2011; Plener et al., 2016), SSRI seems to be the preferred treatment for adolescent MDD with NSSI behavior, because they are beneficial to alleviate depressive symptoms and do not seem to increase the rate of NSSI (Cheung et al., 2018; American Psychological Association [APA], 2019). Based on this, sertraline was selected as drug treatment in this study. The results showed that the frequency of self-injury behavior decreased significantly after the sertraline treatment. Previous studies have different evidence. The results of a study in the group of adolescent refractory depression show that the subjects who choose SSRI for intervention have the lowest incidence of self-injury behavior (Brent et al., 2009). Glenn found that among adolescents with anxiety disorder, the frequency of self-injury in the intervention group combined with fluoxetine decreased significantly compared with adolescents who only used cognitive behavioral therapy (Melvin et al., 2019). However, a recent meta-analysis of psychotropic drugs for the treatment of NSSI in children and adolescents showed that there was no statistically significant difference in the occurrence of NSSI in adolescents between SSRI and the control group (drug or placebo) (Eggart et al., 2022). Whether there is a recommended drug choice for NSSI behavior of adolescents needs to be carried out in a larger randomized controlled study in the future.

The results of regression analysis showed that baseline P3b amplitudes had a significant positive predictive effect on the 8 week NSSI frequency, which indicated that the higher the baseline amplitude, the higher the reduction rate of 8 week NSSI behavior frequency. Many studies suggest that P3 components are related to response inhibition and executive function (Zhang et al., 2021; Egbert et al., 2022; Reed et al., 2022), which indicates that subjects with higher response inhibition and executive function at baseline, the NSSI behavior are more likely to reduce significantly after treatment. The results of a systematic review of neuroimaging of NSSI behavior showed that the activation of brain areas related to executive function decreased in NSSI samples (Brañas et al., 2021). The results of a near infrared spectroscopy study suggest that NSSI patients show the deactivation of the dorsolateral prefrontal cortex (DLPFC), which plays a key role in the executive regulation of cognitive and behavioral responses to the environment (Zahid et al., 2020). The results of a cross-sectional resting state fMRI study in MDD samples of adolescents with NSSI behavior also provide supporting evidence (Huang et al., 2021). In the past, most studies on self-injury behavior focused on the use of scale evaluation to find mediators or regulatory variables, and most studies on neuroimaging were cross-sectional studies. To our knowledge, this study found neurophysiological markers that can predict the reduction of self-injury behavior in adolescent MDD with NSSI for the first time.

This study has some limitations. firstly, the sample size is relative small. Although we found that the baseline P300 index can predict the reduction of adolescent MDD patients self-injury frequency, this needs to be verified in a larger cohort; adolescent MDD group without NSSI behavior was not included in this study, which is also a limitation; 8 weeks follow-up is relatively short, we will continue to follow up.

## Conclusion

Compared with HC subjects, the cognitive impairment of adolescent MDD with NSSI patients was mainly manifested in response inhibition, decreased executive function and poor anti-interference ability. Baseline P300 can be used as a potential predictor of the improvement of 8 week NSSI frequency in MDD adolescents with NSSI behavior.

## Data availability statement

The datasets presented in this article are not readily available because they contain sensitive patient information. The data supporting the conclusions of this article will be made available upon reasonable request by any qualified researcher. Requests to access the datasets should be directed to the corresponding author.

## Ethics statement

The studies involving human participants were reviewed and approved by Ethics Committee of the First Hospital of Shanxi Medical University. Written informed consent to participate in this study was provided by the participants’ legal guardian/next of kin.

## Author contributions

HSL and YW designed the study and involved in data acquisition, analysis, and interpretation. XL and TR contributed to the data acquisition. GL, YX, DQ, and CY contributed to the study design and data interpretation. XZ involved in clinical assessment of participants and involved in patient follow up. ZL served as advisors and were responsible for overall oversight of the study. All authors participated in the drafting or critical review of the article, gave final approval of the version to be published, and agreed to be accountable for all aspects of the work.
